# Wearable Wrist Movement Monitoring Using Dual Surface-Treated Plastic Optical Fibers

**DOI:** 10.3390/ma13153291

**Published:** 2020-07-24

**Authors:** Jing Li, Jian Liu, Cheng Li, Hui Zhang, Yizuo Li

**Affiliations:** 1 School of Smart City, Beijing Union University, Beijing 100101, China; 1365249180@126.com; 2 School of Instrumentation and Optoelectronic Engineering, Beihang University, Beijing 100191, China; 18810927029@163.com (J.L.); huizhang@buaa.edu.cn (H.Z.); 3 Shenzhen Institute of Beihang University, Shenzhen 518063, China

**Keywords:** optical fiber wearable senor, wrist movement detection, etched grating POF, side-polished POF, motion trajectory recovery

## Abstract

Regarding high-sensitivity human wrist joint motion monitoring in exercise rehabilitation; we develop a pair of novel wearable and sensitivity-enhanced plastic optical fiber (POF) strain sensors consisting of an etched grating fiber and a side-polished fiber stitched into a polyamide wrist brace. The two flexible and surface-treated fibers are; respectively; featured with an etched periodic gratings with a pitch of 6 mm and a depth of 0.5 mm and a D-shaped side-polished zone of ~300 µm depth and ~30 mm length; which, correspondingly, show the sensitivities of around 0.0176/° and 0.0167/° in a normalized bending angle by far larger than a conventional commercial POF, because it achieves a more sensitive strain-induced evanescent field interaction with the side-machined fibers. Moreover, in terms of the sensor response to bending deformation in the range of −40°~+40°, the former exhibits a better sensitivity in lower angle change, while the latter is superior as the bending angle increases; thereby arranging the two modified POFs separately at the side and back of the human wrist, in order to decouple the wrist joint behaviors induced by typical flexion-extension or abduction-adduction movements. Then, the circular and pentagonal wrist motion trajectory patterns are investigated, to demonstrate the maximum average single-axis motion error of 2.94° via the transformation of spatial angle to plane coordinate for the fabricated couple of POF sensors, which is lower than a recognized standard of 5°, thus suggesting the great potential in wearable exercise rehabilitation of human joints in the field of medical treatment and healing.

## 1. Introduction

Today, stroke is one of the leading causes of mortality in adults age 15 years and over throughout the world, and the worldwide incidence of stroke is set to escalate from 15.3 million to 23 million by 2030 [1]. Moreover, it is estimated that, following a stroke, only 15% of stroke survivors will gain complete functional recovery for both the upper and lower extremities with walking and mobility being key issues [2]. With regard to the importance of regaining mobility, a number of technological aids have been reported to enhance poststroke motor recovery; however, expensive, large and cumbersome rehabilitation equipment necessitating a therapist will be required [3], which is obviously inappropriate for independent home-based or portable poststroke rehabilitation and motion monitoring. Therefore, recently wearable sensors or devices have been widely utilized for poststroke rehabilitation by monitoring physical activities and corresponding movement conditions, including accelerometers, gyroscopes, magnetometers, fabric and body-worn sensor networks [4], wherein the use of external wearable devices has been proved effective for improving the function of the lower limb in adult stroke survivors, especially for those for whom self-managed interventions may be more appropriate [5].

In rehabilitation training of the upper limb, wrist rehabilitation training is very important for persons after stroke in consideration of the valuable understanding of the relationship between pathological changes and physical disabilities at the wrist [6,7,8]. For example, during a wrist training session, a hemiplegic subject would be required to perform the wrist flexion and extension [8]. Hence, a quantitative assessment of wrist motion behaviors is necessary for evaluating the effectiveness of rehabilitative treatments and the functional impairment of patients. Note that measurements of joint digit range of motion are one of the main quantitative methods of hand-function assessment. Traditionally, mechanical goniometers mounted on each hand joint were used to measure flexion and extension bending angles [9]. However, in addition to the requirements for manual data recording and large size, the traditional goniometric measurement process is tedious and time-consuming for hand therapists and patients. Currently, common devices used for this purpose are electromechanical goniometers, which generally come into operation by using resistive potentiometers or strain gauges [10,11]. Conventional metal foils and semiconductor-based strain gauges are incompetent for use in wearable sensing, due to their poor mechanical compliance and limited workable range [12]. In this case, flexible, sensitive and facile-fabricated strain sensors are highly desirable for the measurement of fast variations of wrist joint movements. Nanomaterials with excellent nanoscale flexibility and electrical properties, such as carbon nanotube, graphene, d, have been recently reported for wearable strain sensors. Unfortunately, these devices face the constraint of the combination of high strain sensitivity and broad sensing range, as well as the incapability of sensing multiple forms of mechanical deformations [13]; thus, a graphene-based composite fiber with “compression spring” architecture was developed to detect human finger bending and knee motions by sensing tensile strain and bending [13]. Though the presence of complicated and laborious fabrication process of the graphene-based fiber involving air plasma treatment, etched ripple-like texture, hydrophilic treatment and multiple dip-coating with graphene oxide, the as-prepared electrical fibers are found to be lightweight and flexible enough to conform to arbitrary curved surfaces.

In reality, in comparison with these electrical counterparts, fiber-optic sensors are increasingly used for human joint monitoring, because of the advantages of small size, light weight, flexibility, versatility and immunity to external electromagnetic interference [14]. Moreover, compared with the fabricated electrical fibers in reference [13], optical fibers can be more easily achieved by a controllable machining process. In general, optical fiber sensors can be implemented using macro-bend loss effect-based intrinsic or fiber tilting angle loss-based extrinsic approach. Several types of optical fibers have been reported and implemented in wearable biomedical sensors include fiber Bragg grating (FBG) [15], side-polished FBG [16], plastic optical fiber (POF) [17], notched side-ablated polymer optical fiber on a fabric substrate [18] and elastomer-based optical fiber with plasmonic gold nanoparticles [19]. Among them, POF wearable sensors are often utilized in detecting bending deformation [17], upper- and lower-limb angular movement [20], respiratory rate [21] and reflective pulse oximetry [22] by means of the intensity attenuation of the luminous wave in the optical fiber, because POF possesses yarn-like flexibility and better coupled power, and can even be incorporated into textiles [19]. However, these conventional POF sensors usually demonstrate low bending sensitivity, especially at lower bending deformation, regardless of the fluctuations in detected power. According to previously published work [14], these external power disturbances related to intensity-based sensors can be compensated for, nonetheless, by various intensity referencing schemes. In order to enhance the sensitivity of a bent POF sensor, a simple side-polishing method is generally adapted, because the mechanical resistance of the fiber allows an easy removal of a portion of the jacket, cladding and core [23]. More importantly, since the fiber core in a D-shaped fiber is asymmetrically located relatively to the geometrical center of the cross-section of the cladding, the optical transmittance attenuation of the side-polished fiber is strongly affected by the bending. For example, Kuang [24] and Bilro et al. [25] presented a study of side-polished POF sensors, applied to curvature and strain measurements in samples subjected to flexural and tensile loading conditions and the human gait, respectively, based on macro bending effects. Although the results showed the feasibility of this type of D-shaped sensor for monitoring bending strain and knee joint movements, only a single-axis joint movement was monitored. By contrast, the wrist joint is capable of implementing three degree-of-freedom movements (flexion-extension, abduction-adduction and rotation), wherein the last one can be decomposed into the former two movements. To the best of our knowledge, few efforts are being made to develop POF sensors for wrist movement monitoring in the field of wrist rehabilitation exercise.

Herein, from the perspective of characterizing bending strain induced by wrist movements, we report a simple, flexible and wearable dual-sensor measurement scheme using a side-polished POF without external coatings and an etched grating POF with periodic cutting grooves, in virtue of the combination of enhanced macro-bend loss and intensity attenuation of the luminous wave transmitting in the optical fiber. This novel scheme can not only offer higher sensitivity to bending deformation than a conventional POF sensor, due to more sensitive strain-induced interactions with the fiber, but also implement more complicated wrist activities, such as wrist motion pattern recognition, compared with single-degree-of-freedom movement only by the side-polished POF sensors, as described earlier. Our previous preliminary work can be referred to for developing this type of POF sensor assembled on a short wristband [26]; however, a simple sensor fabrication and fixing method limits the performance of the developed POF sensor. In this paper, the combination use of a 3D-printed fixture and an optical fiber graver improves the sensor fabrication accuracy. Then, an improved polyamide wrist brace with fixing supports at palm and wrist parts is employed for the POF sensor fixation. Moreover, the measurement principle for wrist bending monitoring by using dual surface-treated POFs is established to increase the amount of evanescent field loss at the sensitive area. In this case, along with the use of an angle turntable for characterizing the sensor sensitivity to the bending angle, wrist circular and pentagonal motion trajectory test reveals the average resultant angle errors of 3.06° and 4.29°, in terms of the regressive normalized angle sensitivity in the corresponding flexion-extension and abduction-adduction ranges of −35°~+35° and −20°~+30°, along with a maximum resultant absolute error of 4.29°, which is comparable to the reliably accepted movement evaluation performance of 5°, according to the American Medical Association [21].

## 2. Sensor Design and Working Principle

### 2.1. Fabrication of the Etched and D-Shaped POF Sensors

**Figure 1:**
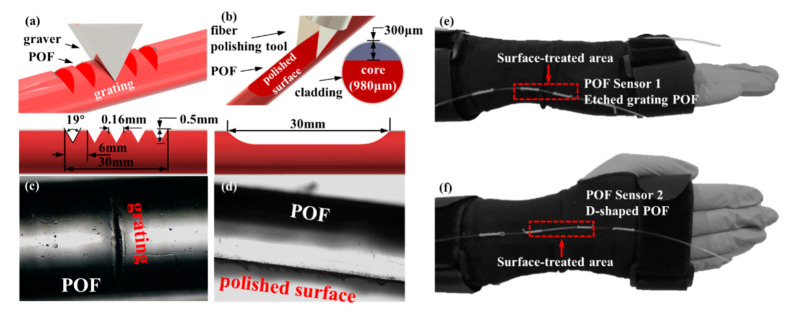
(**a**) Etching periodic grating and (**b**) side-polishing the cladding surface on a plastic optical fiber (POF). Microscope view of (**c**) the etched grating and (**d**) the side-polished fibers. (**e**) Side view and (**f**) front view of a wearable wrist brace with the two surface-treated POFs.

Referring to the schematic fabrication process of the modified POF sensors, as shown in Figure 1a–d, a commercial POF (ESKA-SK40, Mitsubishi, Tokyo Japan), with an outer cladding diameter of 1 mm and a central wavelength of 650 nm, was employed for the subsequent surface machining process. Referring to Figure 1a, the conventional commercial POF was arranged in a 3D-printed acrylonitrile butadiene styrene (ABS) resin fixture with an arc-shaped groove having a same diameter of 1 mm as the fiber. The fixture is featured with the molded grating structure with a pitch of 6 mm, a depth of 0.5 mm and a slot opening of ~19°. Then, with the aid of the molded grating structure of the fixture, the periodic gratings, made of 5 grooves in a grating length of 30 mm, were etched into the POF by a graver. According to Figure 1b, another POF fiber was also fixed in an ABS mounting fixture made by 3D printing technology to fabricate a side-polished fiber with a D-shaped polishing zone of ~300 µm depth and ~30 mm length by using an optical fiber cutting tool. It should be added that the pitch, the shape and especially the depth of the grating would affect the POF sensor sensitivity to bending deformation. Considering this paper is currently focused on a wrist movement monitoring experiment, the influence of these parameters on the attenuation of light in the structurally imperfect areas will be explored in more detail in the further research. Moreover, Figure 1b indicates that the thickness of cladding and diameter of fiber core of the POF are, respectively, 10 µm and 980 µm. The etched groove and side-polished surface of the two POFs are, respectively, depicted in Figure 1c,d. Then, the resulting etched POF and side-polished POF sensors were embedded in a polyamide wrist brace for human wrist joint movement monitoring, as shown in Figure 1e,f. Note that, since the human wrist is a complex series of joints that are formed around the carpal bones and the radius and ulna, the wrist is typically capable of offering the flexion-extension, abduction-adduction and supination-pronation movements. However, the former two items could be combined to form the supination and pronation motions. In other words, both flexion-extension and abduction-adduction behaviors are two basic movements of wrist. Hence, it would be preferable to decouple the aforementioned two wrist movements by the use of appropriate wearable sensors. Due to their distinct bending angle-dependent strain sensitivities caused by optical attentions that are remarkably affected by the bending curvature, the etched grating and the side-polished POFs were respectively arranged at the outside edge and central back of one hand, i.e., the positions proximal to triquetrum bone and scaphoid bone of wrist joint, as illustrated in Figure 1e,f. The response to bending angle for the two movements will be further investigated by the following POF angle measurement in Section 3.

### 2.2. Optical Power Loss in Bent Fiber

Since standard multimode POFs have a high numerical aperture allowing more than two million propagating rays, radiation losses in these fibers are usually calculated through a geometric optic approach [24], wherein the power loss is represented as a function of the curvature radius. In this manuscript, the bending angle-based wrist joint movement POF sensor is primarily based on bending loss, in which the amount of light leaking through the curved portion of the bent fiber is increased. The amount of optical power loss is dependent upon the interaction of the evanescent light with the core-cladding of the optical fiber. In order to explain the measurement principle related to the light propagation loss transmitted in POFs, the influence relation between the power loss and the POF surface structure should be analyzed. Hence, considering the fiber stress through use of the effective bend radius of a POF, the power loss coefficient *ξ* in bent optical fibers is given by [27]

(1) $$\xi =\frac{\pi^{1/2}\kappa^{2}\exp\left(-\frac{2\gamma^{3}R_{\mathrm{eff}}}{3\beta_{z}^{2}}\right)}{2R_{\mathrm{eff}}^{1/2}\gamma^{3/2}V^{2}K_{m-1}\left(\gamma a\right)K_{m+1}\left(\gamma a\right)}$$

where the *K* term is the modified Bessel function; m is the azimuthal mode number (equal half the number of azimuthal zeros); a is the fiber core radius, *R*_eff_ is the effective bending radius of fiber; *β_z_* is the modal propagation constant; *V* is the optical fiber normalized cut-off frequency; *κ* and *γ* respectively represent the field decay rates in the core and cladding. Note that *V*, *κ* and *γ* are respectively defined by

(2) $$\left\{ \begin{array}{l} V=\frac{2\pi a}{\lambda }\sqrt{n_{\mathrm{core}}^{2}-n_{\mathrm{clad}}^{2}}\\ \kappa =\sqrt{k_{\mathrm{core}}^{2}-\beta_{z}^{2}}\\ \gamma =\sqrt{\beta_{z}^{2}-k_{\mathrm{clad}}^{2}} \end{array}\right.$$

where *λ* is the wavelength of light; *n*_core_ and *n*_clad_ is the core and cladding refractive index of fiber, respectively; and *k*_core_ and *k*_clad_ is the wave number in in the core and cladding, respectively.

Since the numerical aperture represents the ability of the fiber to transfer light, the effective numerical aperture NA_eff_ of a bent cylindrical fiber can be expressed by [28]

(3) $${\mathrm{NA}}_{\mathrm{eff}}=\sqrt{n_{\mathrm{core}}^{2}-n_{\mathrm{clad}}^{2}{\left(1+\frac{a}{R_{\mathrm{eff}}}\right)}^{2}}=\sqrt{n_{\mathrm{core}}^{2}-n_{\mathrm{clad}\_\mathrm{eff}}^{2}}$$

where *n*_clad_eff_ is regarded as the effective cladding index, which is written as *n*_clad_(1 + *a*/*R*_eff_). Thus, in terms of Equations (1) and (3), compared with those unprocessed optical fibers, POF is more suitable for human joint motion detection, owing to its high numerical aperture and large core diameter. For a specific POF sensor, the bending loss is only as a function of R_eff_ in the optical fiber. Moreover, the optical power loss induced by the macro-bend effect could be intensified by modifying the parameter *n*_clad_eff_ via structural imperfections on the outer side of fiber. For this reason, an external cladding on a POF was removed up to a part of the core layer in this paper. Although the reduced radius of the imperfect fiber region tends to increase NA_eff_ by referring to Equation (3), the power loss coefficient ξ becomes greater, due to the decreased *a* and *V*, according to Equations (1) and (2). In other words, the modified surface weakens the absorption of light in an equivalent cladding replaced by an air layer, thereby increasing the effective refractive index *n*_clad_eff_ in the polished sensitive region.

In accordance with the bending effect-based displacement measurements by Babchenko et al. [27], in general, the input and output numerical aperture will be the same; however, the output numerical aperture decreases with the increase in a macro-bend curvature of fiber. Additionally, the numerical aperture decreased more when a series of small imperfections, replacing a single large imperfection of the same length, was placed on the fiber. In this way, considering the relatively lower angle range in abduction-adduction movement, an etched grating POF with a periodic multi-structural imperfection was proposed, to enhance the sensitivity to the given wrist movement. As a result, Equations (1)–(3) represent the influence relation between the power loss and the POF sensor structure, which contributes to the structural optimization of the POF sensors, so as to increase the amount of evanescent field loss at the sensitive area of the bent POF. It is worth noting that, since the core and cladding power coupling occurs at all wavelengths in the POF as a multi-mode fiber with several core modes and cladding modes, such a POF sensor. With a periodic grating structure, will cause the coupling of the light power among various guided modes, as well as between guided modes and leaky modes, thereby resulting in light propagation loss [29]. As a result, the developed POF sensor for wrist movement monitoring is appropriate for intensity instead of wavelength modulation schemes.

## 3. Experiment and Analysis

### 3.1. Angle Calibration Experiment of POF Sensor

**Figure 2:**
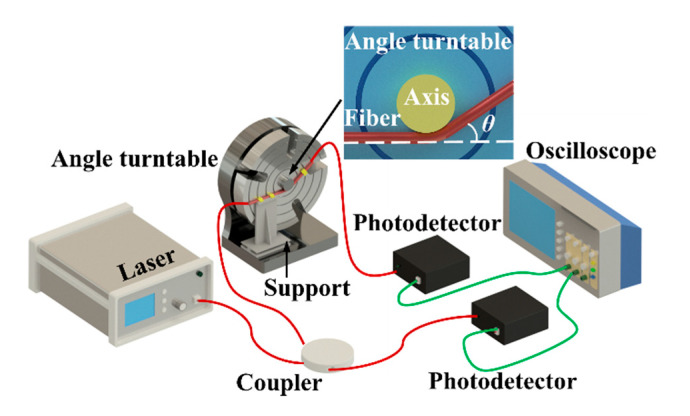
Schematic illustration of angle calibration experiment for the developed POF sensor.

As mentioned above, the two sensitivity-enhanced POF sensors with different surface-treated structures behave different diverse responses to the bending angle. Thus, the angle sensitivity measurement is introduced before wrist movement detection. In order to evaluate the performance of the developed surface-treated POF sensors, a conventional POF was introduced in the angle calibration experiment. In other words, two kinds of POF sensors (a D-shaped POF and an etched grating POF) were fabricated in this manuscript. Figure 2 shows the experimental setup for measuring the bending characteristics of the presented POF sensors, wherein the POF sample (conventional POF, D-shaped POF and etched grating POF) was tested by a turntable with a resolution of 1/60°. A semiconductor laser (QST-650,QST-650, Shenzhen Tycksys Co., Ltd., Guangdong, Shenzhen China) with a wavelength of 650 nm was used to illuminate the POF sample through an optocoupler, and the corresponding transmitted light power was then captured by an oscilloscope (TDS1012C-EDU, Tektronix, Beaverton, Oregon, US), with a sampling frequency of 20 kHz through a 1 MHz bandwidth photodetector (SM-3001, Shenzhen Tycksys Co., Ltd.) with a conditioning amplifier. The optical power stability of the laser with a power output of 1 mW is represented by ±0.05 dB. Despite the lower optical power fluctuation of about ±0.01 mW, an optical fiber circulator connected with a photodetector was utilized for offsetting the light intensity change by monitoring the output power of laser. It should be added that the large-sized commercial laser in the experiment could be replaced with a small on-chip laser diode to implement future wearable applications. Referring to the inset in Figure 2, the POF sample was horizontally arranged below the 40-mm diameter fixed center shaft of the turntable, wherein one side of the sample without a sensitive structure was mounted on a thin plate, and the other side with a sensitive region was fastened on the turntable. Moreover, the side-machined surfaces of the two POFs faced upwards. Thus, the POF sample that is attached on the turntable will bend with the rotation of the turntable, thereby causing macro-bending deformation and then loss of transmitted light intensity. As the angle θ in Figure 2 increases, the loss of transmitted light intensity will reduce because of the orientation of the aforementioned surface-treated fiber facing upwards. In contrast, the loss of transmitted light intensity will increase when the side-machined surfaces of the POFs faced downwards. The two kinds of fiber orientation modes can achieve roughly the opposite characteristic changes. In this way, the dependence of the optical power loss in POF upon rotation angle can be determined for the tested three kinds of POF samples. Note that the power loss in the unthreaded part of the conventional optical fiber without surface treatment is about 150 dB/km.

**Figure 3:**
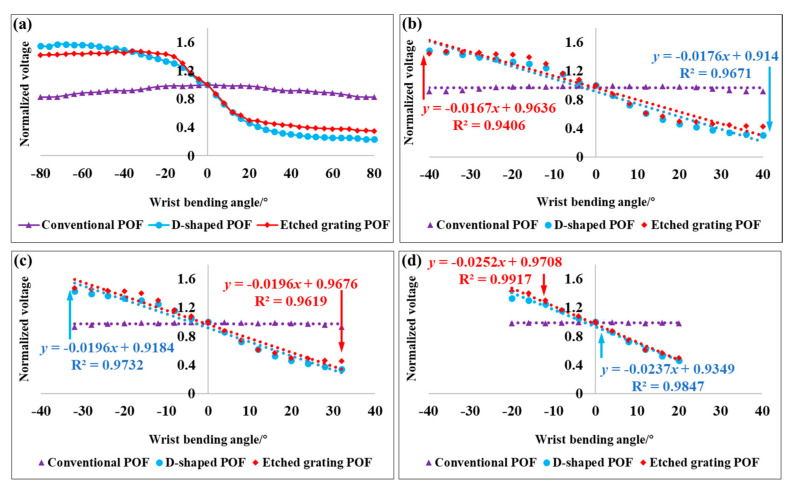
(**a**) The response to bending angle for the POFs with different sensitive structures. The measured normalized sensor output voltage at various angle ranges: (**b**) −40°~+40°, (**c**) −32°~+32° and (**d**) −20°~+20°.

Referring to Figure 2 again, the proposed POF sensor utilizes a simple intensity-modulated scheme to detect the change of rotation angle (*θ*) on the basis of light transmission loss, due to amplified bending effect associated with fiber curvature variations. Note that the angle *θ* ranges from 0°, equivalent to a rest position, to ±80° with an interval of 4°. Note that the positive change in angle indicates a bending to the outside of side-machined surface, while the negative one implies a bending to the inside of side-machined surface. During angle calibration experiments, the turntable will turn back to its rest position (*θ* = 0°) for the clearing of cumulative error whenever it rotates to the limit of the tested angle range. Figure 3 displays normalized photoelectric signal outputs in response to *θ*. The normalized result is defined by the output voltage *V_θ_* at the angle of *θ* divided by the original output voltage *V*_0_ when *θ* = 0°, i.e., *V_θ_*/*V*_0_, so as to eliminate the effect of initial optical power deviations resulted from distinguishing surface structures existing in the three kinds of POF sensors. It needs to be added that *V_θ_* is the light-intensity-corrected output voltage value that is equal to *V*_i_-*V*_dif_, where *V*_i_ is the current sensor output voltage. *V*_dif_ can be determined by *V*_ref_i_-*V*_ref_0_, where *V*_ref_i_ is the current output reference voltage and *V*_ref_0_ is the original value for the output reference voltage through the optocoupler, acting as a reference directly connected with the coupler, as depicted in Figure 2. Similar compensation method was also previously reported in a POF curvature sensor for wearable devices [30]. In view of the relatively simple differential compensation scheme for the light intensity fluctuations caused by light source instability, a power fluctuation compensation scheme for intensity-based POF sensor via the light reflection [31] could be introduced in the following work, to stabilize the light intensity disturbances for further improving the sensor performance. Then, it can be seen from Figure 3a that the conventional POF labeled by the purple line shows a maximum normalized light intensity of 1 at a rest position, while it gradually decreases as the angle *θ* varies in positive or negative directions. This non-monotonic phenomenon means that the conventional POF is an inappropriate option for wrist movement measurement, despite an observable low sensitivity to a bending angle larger than ~±40°. Moreover, the maximum normalized light intensity change was about 0.09 for the conventional POF sensor in the bending angle range of ±80°, which also demonstrated an extremely low light power loss in the unthreaded part of the POF sensor induced by fiber curvature. In contrast, D-shaped and etched grating POFs, denoted by blue and red lines, exhibit more obvious responses whose maximum normalized light intensities approach to around 1.6 and 1.4, respectively. Additionally, their responses are symmetrical with *α*, therefore offering movement direction recognition, which is mainly due to the increase in optical power loss caused by the bending inward towards the palm direction and the decrease caused by the bending outward towards the back of hand. In addition, as the angle gradually increases, the former tends to present higher optical power change. However, they both show a good linear monotonic correlation with the bending deformation within a range of ±40°.

In order to more accurately probe the bending sensitivity that is enhanced using the developed sensors, the angle experiments within various ranges of ±40°, ±32° and ±20° were performed, respectively. By comparison with Figure 3b–d, it can be clearly concluded that the conventional POF is improper for wrist motion monitoring, due to its extremely low angle sensitivity, which is almost zero. The normalized sensitivity of D-shaped POF increased to 0.0196/° and then to 0.0237/° from 0.0176/°, as the angle reduced from the initial ±40° to ±32° and then ±20°, with the corresponding rising rates of 11.4% and 34.7%, along with excellent goodness of fits larger than 96% by using the least squares fitting method. By contrast, the normalized sensitivity of etched grating POF also reached to 0.0196/° and then 0.0252/° from the beginning of 0.0167/°, with great goodness of fits of exceeding 94%; however, the corresponding rising rates distinctly raised by 17.4% and 50.9%, as listed in Table 1. Therefore, it can be inferred that the etched grating POF is superior to the D-shaped POF at a lower bending angle range, but the latter is inclined to have better sensitivity and linearity as the bending angle becomes larger. The phenomenon is primarily due to a more evanescent field interaction with the side-polished fiber surrounded by air, especially when larger fiber curvature variations occur.

### 3.2. Wrist Movement Angle Detection

**Figure 4:**
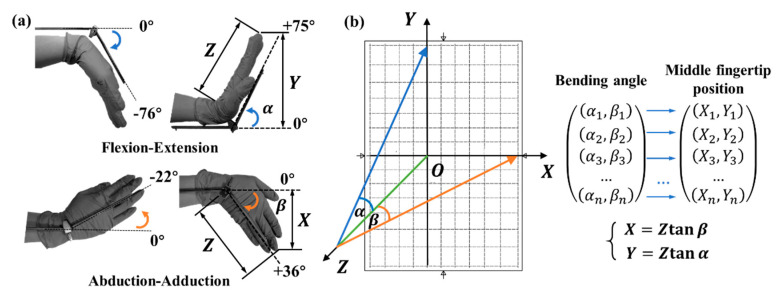
(**a**) Schematic illustration of wrist motion angle detection. (**b**) The transformation of the spatial angle of wrist motion to the plane coordinate (*X*, *Y*).

In terms of the measured performance of the developed surface-treated POF sensors, wrist movement angle detection and wrist movement trajectory detection were performed. In these cases, as mentioned above, the two surface-treated POF sensors were, respectively, arranged at the outside edge and central back of one hand, i.e., the positions proximal to triquetrum bone and scaphoid bone of wrist joint. The sensitive region in the former fiber faces inwards, while the one in the latter fiber faces upwards. Other experimental devices used for these two wrist movement measurements are same as those in Figure 2. As shown in Figure 4a, the flexion-extension and abduction-adduction movements can provide the maximum angle ranges of −76° to +75° and −22° to +36°, respectively [32]. However, the actual angles for implementing ordinary flexion-extension and abduction-adduction rehabilitation training for stroke patients are generally limited in the narrow ranges of −5° to +30° and −10° to +15° [33]. That is to say, the flexion-extension movement is required to offer a relatively wider bending range, compared with the abduction-adduction one. Hence, a compromise between a larger range of bending angles and a better sensitivity/linearity was established in consideration of different sensitivities to bending angle. Then, based on the measured responses featured by the two fabricated POFs, the D-shaped POF was arranged at the central position of the back of hand via a polyamide wrist brace, in order to sense the flexion-extension movement with a wide angle of motion, and then the other etched grating POF was fixed at the outside edge of the wrist brace, as illustrated in Figure 1e,f. Then, for the sake of effectively evaluating spatial motions of wrist, the transformation of the spatial angle of a certain wrist bending motion to the plane coordinate (*X*, *Y*) was established as given in Figure 4b, wherein the graph’s horizontal X-axis shows the movement position corresponding to abduction-adduction movement, and, similarly, the vertical Y-axis corresponds to the flexion-extension movement position.

Assuming the distance between the wrist joint of a tester and the coordinate plane is *Z*. For simplification, the finger joints are keep straight, and the hand equipped with dual POF sensors moves around the wrist joint but holds a fixed position relative to the *X*-*Y* plane. In this case, *Z* is known for a certain tested object. In terms of the trigonometrical function relation, the coordinate (*X*, *Y*) can be achieved by

(4) $$\{\begin{array}{l} X=Z\tan\beta \\ Y=Z\tan\alpha \end{array}$$

where *α* and *β* are the angles between the Z-axis and the movement positions of hand in the Y-axis and the X-axis, respectively. In this way, with the aid of Equation (4) and the given coordinate (*X*, *Y*), the bending angle for calibrating wrist movement can be determined.

**Figure 5:**
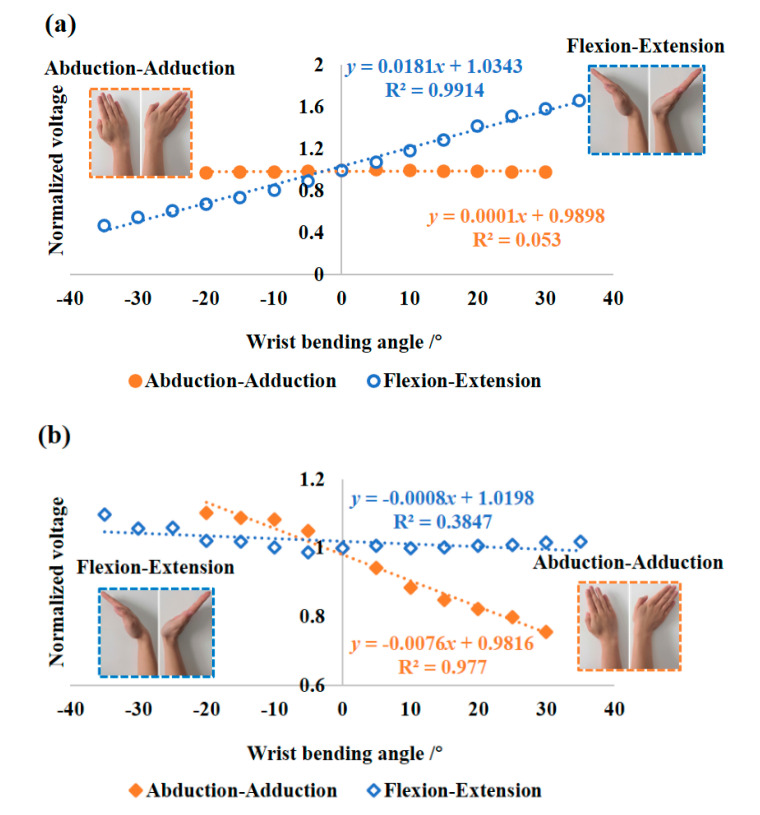
The measured responses to wrist flexion-extension and abduction-adduction movements for (**a**) the side-polished POF and (**b**) the etched grating POF.

In view of the limited ranges of −5°~+30° and −10°~+15° for the general flexion-extension and abduction-adduction rehabilitation training mentioned above, the wider scopes of −35°~+35° and −20°~+30° for the two types of wrist movements were correspondingly investigated in wrist motion angle experiments. At the beginning, a 26-year-old male tester wearing the wrist brace kept his hand horizontally, i.e., perpendicular to the *X*-*Y* plane formed by a rigid plate marked with *X* and *Y* scales. Meanwhile, the middle fingertip of his hand to be tested was contacted with the origin (*O*) of coordinates, which means *Z* = 19 cm in consideration of the length of 19 cm between the wrist joint of the tester and his middle fingertip. To estimate the cross-sensitivity to flexion-extension and abduction-adduction motions for the designed D-shaped and etched grating POF sensors, the wrist was supposed to move firstly along the single X-axis and then Y-axis with an angle interval of 5°, respectively. Therefore, only wrist flexion-extension and abduction-adduction movement modes will be recorded, as revealed in Figure 5.

It can be seen in Figure 5a that the side-polished D-shaped POF was insensitive to the abduction-adduction movement; however, it exhibited a normalized angle sensitivity of 0.018/° (R^2^ = 0.991) for the flexion-extension movement in the tested angle range, which can be approximated as:

(5) $$N_{D-\mathrm{POF}}=0.018\alpha +1.034$$

where *N*_D-POF_ is the normalized voltage output for the D-shaped POF sensor.

On the contrary, in Figure 5b, the etched grating POF showed a nearly zero response in the tested angle range, where even the weak and negligible response as a whole appeared at larger bending angles; however, the etched grating POF showed an improved normalized angle sensitivity of 0.008/° (R^2^ = 0.977) in response to the abduction-adduction movement, which can be written as:

(6) $$N_{E-\mathrm{POF}}=-0.008\beta +0.982$$

where *N*_E-POF_ is the normalized voltage output for the etched grating POF sensor.

The two distinct sensitive behaviors can be primarily explained by the fact that the polished surface of D-shaped POF mounted in the wrist brace is parallel to the palm plane, thus, causing remarkable optical power changes in the flexion-extension movement direction. Conversely, the grating, which was mechanically cut in a POF in a direction perpendicular to the axis of the fiber, is also perpendicular to the palm plane, therefore leading to dominating optical power changes in the abduction-adduction movement direction, and certain observable changes at wider angles for the flexion-extension motion. However, it is worth emphasizing that, due to their distinct bending angle-dependent strain sensitivities caused by optical attentions that are remarkably affected by the bending curvature, the etched grating and the side-polished POFs were, respectively, arranged at the outside edge and central back of one hand, i.e., the positions proximal to triquetrum bone and scaphoid bone of wrist joint. Thus, the two kinds of POF sensors arranged on the wrist brace are able to sense the corresponding wrist motions. As a result, based on the determined aforementioned sensitivities to bending angle for the two sensors, different wrist bending angles can be discriminated by the two kinds of POF sensors.

Additionally, it is important to add that, in comparison to the measured angle sensitivities in Table 1, the normalized sensitivity of ~0.018/° in the range of ±35° for the D-shaped POF in Figure 5a fell in between 0.0176/° and 0.0196/°, in good agreement with the result confirmed by the use of a turntable. Nevertheless, the etched grating POF in Figure 5b achieved an effective normalized sensitivity of ~0.008/° in the angle ranging from −20° to +30°, which is beyond the calibrated threshold range of 0.0196/°~0.0252/°, corresponding to the angle scopes of ±32°and ±20° in Table 1. The reduced sensitivity is partly due to the existence of relative movement induced by the fascia and bone of wrist, instead of simple bending of hand around wrist joint without deformation [34]. Another reason is possibly that the fiber sensitive surface deflection and unstable sensor fixation caused by the stitching connection of POF with wrist brace. Hence, further research on the positioning and mounting technology of the POF sensor on a wearable glove, such as woven optical fibers and flexible fixed mounts, is needed to optimize the dynamic sensitivity of POF sensor in actual wearable applications. As a result, despite a relatively lower sensitivity (~0.018/°) to the abduction-adduction movement than the expected value for the etched grating POF, wrist motion behaviors could be well decoupled by coordinating the use of the two types of designed POF sensors, in combination with the corresponding appropriate mounting positions in wrist brace.

### 3.3. Wrist Movement Trajectory Detection

**Figure 6:**
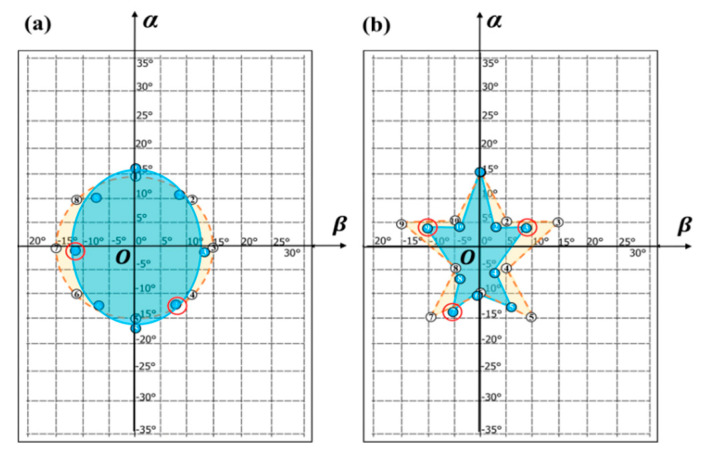
The extracted wrist (**a**) circular and (**b**) pentagonal motion trajectory patterns for the developed wearable sensor, where yellow dashed line and blue solid line represent the predetermined and recovered patterns, respectively.

To further intuitively evaluate the presented dual POF sensors in wrist spatial movement detection, simple graphs, such as round and pentagon, were drawn by wrist postures of a tester. Circular and pentagonal patterns represent the arc-shaped and broken line-shaped trajectories of wrist joint, respectively. Thus, the two patterns can be used to comparatively evaluate the influence of bending angle variation on the stability and sensitivity of the sensor assembly in different wrist motion modes made up of typical line-shaped and arc-shaped movements. Then, according to the recovered graphs, three degree-of-freedom movements of wrist joint for the developed optical fiber wearable sensor can be effectively investigated. As illustrated in Figure 6, the predetermined circular and pentagonal wrist motion trajectory patterns were denoted by yellow dashed lines, on which eight and 10 discrete measurement points concerned with wrist joint compound motions were, respectively, labeled on a rigid plate marked with *α* and *β* coordinate scales. It is worth noting that the position coordinate (*X*, *Y*) in Figure 4b can be transformed to the angle coordinate (*α*, *β*) in Figure 6, on the basis of Equation (4). In this case, these discrete angle measurement points are set in the *α*-*β* plane. Then, in the initial state, the middle fingertip of a tester’s hand was made to contact with the origin (*O*) of the coordinates, while keeping horizontal. At this point, the initial output voltage *V*_0_ for each of the two POF sensors was sampled. Regarding the predefined eight or 10 angle measurement points, the sensor output voltage *V_θ_* was collected one by one in a clockwise direction via dual channel synchronous acquisition. The experiments were repeated three times. In terms of the calibrated relationship between normalized voltage output and bending angle as given in Equations (5) and (6), the bending angles *α* and *β* can be solved by the measured averaged values (*N*_D-POF_ or *N*_E-POF_) at each set angle points. Then a least-square polynomial fitting method was used to recover the graphs from the extracted angle data, as depicted by blue solid lines in Figure 6.

It can be seen from Figure 6 that the extracted graphs roughly conform well to the original ones, although there are still partial deviations at certain points, especially at those points representing abduction-adduction movements at larger bending angles. For this purpose of clearly locating the source of the problem, the calculated angle absolute errors are listed in Table 2, wherein the conformity of simple circular shape is better than that of relatively complicated pentagonal shape in terms of the achieved average resultant angle errors of 3.06° and 4.29° for the two shapes. However, these bigger resultant errors mostly resulted from the abduction-adduction movement-dependent *β* values occurring at increasing points of bending, such as the points “4” and “7” and the points “3”, “7” and “9” marked by the red circles in Figure 6a,b, respectively. As described earlier, this problem can, in part, be elucidated by the fact the fabricated POF sensors, sewn into a wrist brace, were insufficiently stable to allow accurate measurement of bending angles induced by wrist activities. Hence, owing to its relatively complete circular profile of fiber, the sewn etched grating POF is easier to roll with a small angle, particularly performing wrist rotation activity comprising larger abduction-adduction movement, thereby leading to the dislocations of sensitive areas and then dominating discrepancies. More importantly, in addition to the introduced major *β*-angle error, the narrower light leak region would sacrifice the sensor sensitivity in *β*-angle direction as a consequence of the dislocations of sensitive etched areas, accordingly causing a reduced curve profile in the same direction, as demonstrated by the blue areas in Figure 6. In addition, due to the absence of the mounting bracket supporting the upper arm during wrist motions, the position offset of wrist joint will result in additional *α*-angle change to make middle finger pointing to the set measurement azimuth, which has a weak effect on the *β*-angle change on account of the generated translation of wrist. This also explained the reason for higher angle errors in the flexion-extension movement instead of abduction-adduction movement, as shown by the points “1” and “5” for the circular pattern and the point “1” for the pentagonal pattern in Table 2. The average single-axis angle errors for the restored graphs were confirmed as the range of 1.56°~2.94°, with a maximum resultant absolute error of 4.29°. Then, according to the t-test of coefficient significance, the 95% confidence intervals (α = 0.05) on the measured angle errors are also given in Table 2. The resulting average errors, which are lower than the commonly accepted joint movement evaluation performance of 5° [21], are comparable to the measured average errors of between 4.76° and 6.17° in metacarpal and proximal interphalangeal joint angle [35], finger joint angle [9] and wrist movement angle [36].

It should be indicated that the error evaluation scheme using coordinate transformation will be mainly affected by the hand shapes and palm movements of a tester during experiments. In contrast, the use of a miniature and flexible strain sensor embedded in the wrist brace would be superior for providing a reference bending angle with high accuracy. In particular, the solutions involving a robot hand or multiple testers would be superior and could then be introduced in the following work, along with a statistical evaluation technique. In addition, it is well known that the thermo-optic coefficient of the POF is an order of magnitude higher than that of the single-mode glass optical fiber. Therefore, the temperature will influence the POF sensor response. Previous research showed that the power loss per unit temperature in POF is closely related with fiber curvature radius and turns [37]. Since the sensitive fiber fastened to the wrist brace has a curvature radius larger than 20 mm with 1/4 turns, the power loss per unit temperature in the developed POF is approximately lower than 0.003 dB/°C. Note that wrist movement experiments were performed at room temperature. Thus, the temperature fluctuation would exhibit a weak effect on measurement results. To sum up, in combination with data regression correction, further research on the structural optimization, reliable fixation and array technology of the side-machined POF sensors is needed to monitor complicated human joint motions with high sensitivity to wide-range bending deformation, therefore, advancing a real test for a patient with physical disabilities at the wrist.

## 4. Conclusions

Two types of simple, flexible and sensitivity-enhanced POF wearable sensors were demonstrated by side-polishing or etching periodic gratings on the external surface of a POF, respectively. In comparison with the conventional POF, which is nearly insensitive to the normalized bending angle, the fabricated D-shaped and etched grating POF sensors both exhibited remarkably higher normalized angle sensitivities in the tested angle range, because of intensified optical evanescent field interaction with the surface-machined fibers. Moreover, with the decrease in the angle range, the latter showed an increased sensitivity. Regarding the different characteristics in response to bending deformation, the etched grating and D-shaped POFs were, respectively, arranged at the outside edge and central back of hand via a polyamide wrist brace, in order to selectively sense flexion-extension and abduction-adduction movements, due to having achieved extremely low cross-sensitivity to the two kinds of wrist motions. The wide scopes of −35°~+35° and −20°~+30° were investigated in wrist single-axis motion experiments, which, respectively, obtained the normalized sensitivities of 0.018/° and 0.008/° for the aforementioned two POF sensors. Then, the circular and pentagonal pattern-based wrist movement trajectory detection demonstrated the roughly good conformity with the predetermined graphs with a maximum resultant absolute error of 4.29°, along with the average single-axis angle errors ranging from 1.56° to 2.94°, corresponding to flexion-extension and abduction-adduction activities. The resulting composite error lower than the commonly accepted joint movement evaluation performance of 5° verified that the as-fabricated flexible wearable sensor scheme could be extended for applications of virtual reality and healthcare, in addition to exercise rehabilitation.

## Figures and Tables

**Table 1:** The solved normalized angle sensitivity and corresponding goodness of fit at various angle ranges.

Sensor Type	−40°~+40°	−32°~+32°	−20°~+20°
Conventional POF	0	0	0
D-shaped POF	0.0176/° (R^2^ = 0.967)	0.0196/° (R^2^ = 0.973)	0.0237/° (R^2^ = 0.985)
Etched grating POF	0.0167/° (R^2^ = 0.941)	0.0196/° (R^2^ = 0.962)	0.0252/° (R^2^ = 0.992)

**Table 2:** The resolved absolute error of wrist motion-dependent angle coordinate (*α*, *β*).

Point	Round				Pentagram		
	Δ*α* (°)		Δ*β* (°)	$\sqrt{{\left(\Delta \alpha \right)}^{2}+{\left(\Delta \beta \right)}^{2}}$ (°)	Δ*α* (°)	Δ*β* (°)	$\sqrt{{\left(\Delta \alpha \right)}^{2}+{\left(\Delta \beta \right)}^{2}}\;$ (°)
1	1.08		0.38	1.14	3.92	0.85	4.01
2	0.95		−1.61	1.87	−2.12	−1.93	2.87
3	−1.35		−2.32	2.68	−2.25	−5.96	6.37
4	−3.06		−2.87	4.19	−2.42	−2.38	3.39
5	−2.52		0.39	2.55	0.63	−4.45	4.49
6	−2.44		2.69	3.63	−2.11	−0.37	2.14
7	−0.74		4.87	4.92	1.72	4.81	5.11
8	0.36		3.50	3.52	−4.89	1.01	4.99
9	/		/	/	−2.66	5.66	6.25
10	/		/	/	−2.56	1.99	3.24
$\frac{\sum_{i=1}^{N}\left|x\right|}{N}$	1.56		2.33	3.06	2.53	2.94	4.29
Confidence interval (95%)	Lower	0.76	1.08	2.05	1.71	1.47	3.29
	Upper	2.36	3.57	4.07	3.35	4.41	5.28

*x* = Δ*α* or Δ*β*; *N* = 8 for round and *N* = 10 for pentagram.
